# Characterization and mechanism of action of amphibian-derived wound-healing-promoting peptides

**DOI:** 10.3389/fcell.2023.1219427

**Published:** 2023-06-15

**Authors:** Xiakun Wang, Hongcheng Duan, Min Li, Wei Xu, Lin Wei

**Affiliations:** Jiangsu Key Laboratory of Infection and Immunity, Institutes of Biology and Medical Sciences, Soochow University, Suzhou, Jiangsu, China

**Keywords:** amphibian, frog, salamander, wound-healing-promoting peptide, antimicrobial peptide

## Abstract

Wound-healing-promoting peptides are excellent candidates for developing wound-healing agents due to their small size and low production cost. Amphibians are one of the major sources of bioactive peptides, including wound-healing-promoting peptides. So far, a series of wound-healing-promoting peptides have been characterized from amphibians. We hereby summarized the amphibian-derived wound-healing-promoting peptides and their mechanism of action. Among these peptides, two peptides (tylotoin and TK-CATH) were characterized from salamanders, and twenty five peptides were characterized from frogs. These peptides generally have small sizes with 5–80 amino acid residues, nine peptides (tiger17, cathelicidin-NV, cathelicidin-DM, OM-LV20, brevinin-2Ta, brevinin-2PN, tylotoin, Bv8-AJ, and RL-QN15) have intramolecular disulfide bonds, seven peptides (temporin A, temporin B, esculentin-1a, tiger17, Pse-T2, DMS-PS2, FW-1, and FW-2) are amidated at the C-terminus, and the others are linear peptides without modifications. They all efficiently accelerated the healing of skin wounds or photodamage in mice or rats. They selectively promoted the proliferation and migration of keratinocytes and fibroblasts, recruited neutrophils and macrophages to wounds, and regulated the immune response of neutrophils and macrophages in wounds, which were essential for wound healing. Interestingly, MSI-1, Pse-T2, cathelicidin-DM, brevinin-2Ta, brevinin-2PN, and DMS-PS2 were just antimicrobial peptides, but they also significantly promoted the healing of infected wounds by clearing off bacteria. Considering the small size, high efficiency, and definite mechanism, amphibian-derived wound-healing-promoting peptides might be excellent candidates for developing novel wound-healing-promoting agents in future.

## 1 Introduction

Skin, including the epidermis and dermis, can form a protective shield from external physical and chemical environmental challenges (Gallo and Hooper, 2012). As a protective barrier against a range of external stress factors, the skin is the most frequently injured one among all tissues (Martin, 1997; Liu et al., 2014a; Eming et al., 2014). Wound healing is essential for all organisms to survive, and for skin, it developed a set of complex mechanisms to protect itself and to restore tissue integrity when damaged (Mangoni et al., 2016). Efficient wound healing requires the coordinated responses of various cell types within an injured tissue (Enyedi and Niethammer, 2015). Traditionally, acute wound healing includes four overlapping phases known as hemostasis, inflammation, cellular proliferation, and remodeling (Greaves et al., 2013). Following surgery, accidental injury, burns, microbial infection, skin diseases, or metabolic dysfunction, the skin barrier is broken and gives rise to a wound (Kujath and Kujath, 2010). Under normal physiological conditions, restoration of a functional epidermal barrier is highly efficient (Akita, 2019), but the failure of the mechanisms underlying the recovery of damaged tissues can lead to the formation of non-healing chronic wounds (Mustoe, 2004; Demidova-Rice et al., 2012). However, due to lack of therapies for treating non-healing wounds or for speeding up the repair of acute wounds, the number of patients suffering from chronic wounds and impaired healing conditions is reaching epidemic proportions and becoming more burdensome in both human health and economic terms (Sen et al., 2009). In the past decades, a few of growth factors has been developed for wound-healing therapies, including epidermal growth factor (EGF), fibroblast growth factor 2 (FGF-2), vascular endothelial growth factor (VEGF), platelet-derived growth factor (PDGF), keratinocyte growth factor-1 (KGF-1), granulocyte-macrophage colony-stimulating factor (GM-CSF), and granulocyte colony-stimulating factor (G-CSF) (13). However, because of the large sizes corresponding to higher production costs, the therapies based on growth factors have not yet proven to be widespread in clinical application (Julier et al., 2017; He et al., 2019). Therefore, it is necessary to develop efficient drugs for curing chronic wounds and impaired healing. Recently, immunomodulatory peptides with small size and potent wound-healing-promoting activities are becoming attractive candidates for treatment of wounds (Mansour et al., 2014; Hancock et al., 2016).

Amphibians are directly exposed to different environments and their skins interact with kinds of environmental factors, such as microorganisms, parasites, predators, and physical factors (Xu and Lai, 2015). For everyday survival, amphibian skins possess excellent wound-healing ability and represent a resource for prospective wound-healing-promoting compounds (Liu et al., 2014b). So far, a series of wound-healing-promoting peptides have been characterized from amphibians. In addition to amphibian-derived wound-healing-promoting peptides, antimicrobial peptides (AMPs) are expressed diversely and abundantly in amphibian skins as the first line in defense against microorganism infection. For the phases of wound healing mentioned previously, in theory, all peptides with a positive effect on inflammation, proliferation, and remodeling may have the wound-healing-promoting ability (Xu and Lai, 2015). Considering the immunomodulatory properties of AMPs on cell migration, survival, proliferation, and induction of antimicrobial and immune mediators, the AMPs are also a huge pool of wound-healing-promoting peptides (Bernard and Gallo, 2011; Niyonsaba et al., 2017). As we know, AMP expression can be induced by infection. Interestingly, AMP expression can also be induced by injury, which indicates that AMPs may act as multifunctional effector molecules in the anti-infective and repair response by triggering cellular responses. The skins of anuran (frogs and toads) amphibians are an extraordinarily rich source of AMPs (Rinaldi, 2002; Ladram and Nicolas, 2016). When the amphibian is stressed or injured, the peptides stored in skin granular glands can be released in high concentrations into skin secretions, including AMP and wound-healing peptides. In this brief review, we summed up the peptides with the wound-healing-promoting effect derived from salamanders and frogs, many of which can also be classified into AMPs. It suggests that AMPs from frogs can be used as a reserve for screening wound-healing-promoting peptides.

## 2 Wound-healing-promoting peptides from amphibians

### 2.1 AH90

AH90 is a peptide composed of 24 amino acid residues characterized from the frog *Odorrana grahami* (Liu et al., 2014a). It was shown to promote wound healing by inducing cell migration, granulation tissue contraction, and fast re-epithelialization process. All the promoting processes induced by AH90 were attributed to the induction of transforming growth factor β1 (TGF-β1) production in macrophages, and AH90 has a strong capacity to stimulate TGF-β1 secretion. TGF-β is a key factor involved in every phase of wound healing. TGF-β was shown to stimulate keratinocyte migration, angiogenesis, fibroblast proliferation, myofibroblast differentiation, and matrix deposition (Werner and Grose, 2003). So, AH90 has a strong capacity to promote wound healing by stimulating TGF-β1 production in macrophages.

### 2.2 CW49

CW49 is a peptide composed of 11 amino acid residues characterized from the frog *Odorrana grahami* with potential wound-healing ability (Liu et al., 2014b). It was primarily identified by running an angiogenesis screening from a number of small peptides that might have various biological activities. The peptide CW49 showed the most outstanding pro-angiogenic ability among all of the tested peptides (Liu et al., 2014b). Angiogenesis plays a critical role in normal wound healing. However, impaired angiogenesis is often accompanied with some impaired wound healings, such as in diabetic wounds. In addition, many drugs effective in treating the chronic wounds of animal models possess the potent of pro-angiogenic ability, such as VEGF and LL-37 (Koczulla et al., 2003; Galiano et al., 2004; Carretero et al., 2008). CW49 was shown to enhance wound healing in normal and diabetic mice by promoting angiogenesis (Liu et al., 2014b). In addition, preventing an excessive inflammatory response was another underlying mechanism of CW49 in promoting the diabetic wound-healing process.

### 2.3 Temporins A and B

Temporin A (Ta) and temporin B (Tb) are short, linear, and mildly cationic AMPs with 13 amino acid residues identified from the frog *Rana temporaria* (Di Grazia et al., 2014). Both peptides efficiently induced HaCaT cell migration by promoting HaCaT cell proliferation via the EGFR signaling pathway. In addition, Ta was found to chemoattract monocytes by using formyl peptide receptor-like 1 as a receptor (Chen et al., 2004). In addition, their antimicrobial efficacy also played an important role in the infection stage of wound healing. In particular, they could kill *Staphylococcus aureus* and multi-drug-resistant *S. aureus* (MRSA) strains, which could be internalized by human epidermal cells. Furthermore, their small sizes should allow a low production cost, which provides the possibility for their wide application.

### 2.4 Esculentin-1a (1-21) NH_2_

Esculentin-1a (1-21) NH_2_ is a derivative of the AMP esculentin-1a, which was isolated from the skin of *Pelophylax lessonae/ridibundus* (formerly known as *Rana esculenta*) and possessed a wide spectrum of antimicrobial activities with high efficacy against *Pseudomonas aeruginosa* (Di Grazia et al., 2015). The authors showed that all-L but not all-D Esc(1-21) peptide significantly stimulated keratinocyte (HaCaT cells) migration, rather than keratinocyte proliferation to promote re-epithelialization of wound healing (Di Grazia et al., 2015). In addition, the research indicated that Esc (1-21)-promoted HaCaT cell migration required EGFR activation and involved STAT3 phosphorylation, which is similar to LL-37 (Tokumaru et al., 2005). However, all-L Esc(1-21) was more efficient than LL-37 in promoting migration of HaCaT cells *in vitro* and exhibits a wider “therapeutic” window. As described in previous research, EGFR-activated cell migration is predominantly mediated by the STAT pathway in keratinocytes, and STAT3 signaling played an essential role in skin remodeling and wound healing (Sano et al., 1999; Andl et al., 2004). Furthermore, Esc(1-21) preserved the capability of antibacterial activity even at high salt concentrations (Luca et al., 2013) as well as in the presence of serum and tears (Kolar et al., 2015). This significant property was absent in the key human AMPs, such as hBD-2 and LL-37, the antimicrobial efficacy of which was completely lost at the high ionic strength (Bals et al., 1998) or drastically reduced in biological fluids (Huang et al., 2007). The antimicrobial ability of Esc(1-21) can kill microbes, without harming mammalian cells (Luca et al., 2013; Kolar et al., 2015), which makes Esc(1-21) a particularly attractive candidate for wound-healing promoter, especially in the management of chronic, often *Pseudomonas* super-infected, human skin ulcers (Wolcott and Dowd, 2008; Percival et al., 2012).

### 2.5 Tiger17

Tiger17 was synthesized based on tigerinins, which was identified from the skin secretions of frog *Fejervarya cancrivora* (Tang et al., 2014). Tiger17 significantly promoted the proliferation of keratinocytes (HaCaT cells) and human skin fibroblasts (HSFs) in a concentration-dependent manner. An *in vitro* cell scratch assay showed that tiger17 significantly increased the migration rate of keratinocytes. A full-thickness skin wound-healing model confirmed that tiger17 accelerated the healing of full-thickness wounds in mice. The histological analysis revealed that tiger17 promoted regeneration of neo-epidermal tissues and restoration of dermal tissues in the wound of mice. In addition, immunohistochemical analysis indicated that many macrophages were recruited to the unwounded skin margin closed to the neo-epidermis and neo-dermis in tiger17-treated skin wound. In addition, the productions of TGF-β1 and interleukin-6 (IL-6) were significantly increased by tiger17, and the induced expression of TGF-β1 significantly promoted fibroblast-to-myofibroblast transition, which played important roles in wound healing (Haroon et al., 2000; Tredget et al., 2005; Luckett-Chastain and Gallucci, 2009).

### 2.6 MSI-1

MSI-1 was truncated from the N-terminus of pexiganan (Ma et al., 2020). Pexiganan is a synthetic analog of magainin 2 isolated from *Xenopus laevis*, and pexiganan was primarily engineered with high potency against many bacteria (Lamb and Wiseman, 1998; Ge et al., 1999; Ge et al., 2002; Lipsky et al., 2008; Wong et al., 2009; Ma et al., 2020). *In vitro*, pexiganan had an antimicrobial activity against most clinical bacterial isolates cultured from infected diabetic foot ulcers (Fuchs et al., 1998; Ge et al., 1999; Ge et al., 2002). In the study, the application of pexiganan in clinical trials showed that, for mildly infected diabetic foot ulcers, topical pexiganan was clinically comparable to an oral antibiotic (Lipsky et al., 2008). However, for its erythrolysis and poor biological stability, it was not more effective than already approved treatments for diabetic foot ulcers (Lavery et al., 2007). Therefore, a series of peptides were designed by truncating N-terminus of pexiganan (Ma et al., 2020). Among the designed peptides, MSI-1 exerted rapid bactericidal activity and low hemolysis.

Interestingly, MSI-1 efficiently improved the survival rate and wound closure in penicillin-resistant *Escherichia coli*-infected mice by eliminating bacterial counts in mouse organs or subeschar (Ma et al., 2020). In addition, MSI-1 strongly reduced the expression of tumor necrosis factor-α (TNF-α) and IL-6 in skin lesion homogenate but not in serum. Moreover, hematoxylin and eosin (H&E) staining showed that the granulation tissues were much thicker in the MSI-1 (10 mg/kg) group, indicating that the congestion and inflammatory cell infiltration could also be attenuated by the peptide (Ma et al., 2020). So, MSI-1 significantly improved wound closure in the *E. coli*-infected burn-wound model by decreasing bacterial counts and inhibiting the bacterium from spreading systemically to relieve the systemic inflammatory response induced by local infection (Ma et al., 2020).

### 2.7 Cathelicidin-OA1

Cathelicidin-OA1 was identified from the skin of *Odorrana andersonii*, which was a member of cathelicidin AMPs (Cao et al., 2018). Unlike other cathelicidins, cathelicidin-OA1 had no direct antimicrobial activity, acute toxicity, and hemolytic activity, but it did exhibit antioxidant activity. Interestingly, cathelicidin-OA1 significantly accelerated wound healing by inducing HaCaT cell proliferation and HSF cell migration as well as accelerating re-epithelialization and granulation tissue formation by enhancing the recruitment of macrophages to the wound site.

### 2.8 Cathelicidin-NV and antioxidin-NV

Another cathelicidin family peptide, named cathelicidin-NV, was identified from the frog skin of *Nanorana ventripunctata* (Wu et al., 2018). Cathelicidin-NV significantly promoted wound healing in a full-thickness dermal wound model in mice. The *in vitro* assay showed that cathelicidin-NV accelerated re-epithelialization by enhancing the proliferation of keratinocytes and fibroblasts and migration of keratinocytes. Cathelicidin-NV also induced the differentiation of fibroblasts to myofibroblasts and collagen production in fibroblasts, which played important roles in wound contraction and repair processes. Furthermore, all the aforementioned wound-healing processes including migration, proliferation, and differentiation were in need of some factors to drive. In this study, the release of essential factors such as monocyte chemoattractant protein-1 (MCP-1), TNF-α, VEGF, and TGF-β1 were proved to be induced by cathelicidin-NV both *in vivo* and *in vitro*. In addition, cathelicidin-NV showed no cytotoxicity and hemolytic activity toward mammalian cells, and cathelicidin-NV did not have any antimicrobial activities against the tested bacterial strains at a relatively high concentration.

Antioxidin-NV was another peptide with wound-healing-promoting activity isolated from the skin from *N. ventripunctata* (Feng et al., 2021). Antioxidin-NV could rapidly clear reactive oxygen species induced by UVB irradiation, attenuate DNA damage by inhibiting p-histone H2A.X expression, reduce cell apoptosis via inhibiting the expression of cleaved caspase 3, decrease inflammatory response by inhibiting Toll-like receptor 4 (TLR4)/p38/JNK/NF-κB signaling pathway, and finally, provide protection against skin photoaging induced by UVB irradiation.

### 2.9 Cathelicidin-DM

Cathelicidin-DM was also a cathelicidin family peptide, which was isolated from the skin of *Duttaphrynus melanostictus* (Shi et al., 2020). *In vitro*, it efficiently promoted HaCaT, HSF, and HUVEC proliferation and induced HSF, HUVEC, and RAW.264.7 cell migration. In addition, cathelicidin-DM significantly activated the MAPK signaling pathway, including inducing the phosphorylation of ERK, JNK, and p38. *In vivo*, it accelerated the healing of non-infected skin wounds in mice by enhancing re-epithelialization, granulation tissue formation, α-SMA expression, and collagen I deposition. Due to its powerful antimicrobial activity, cathelicidin-DM also effectively accelerated the healing of infected skin wounds in mice (Shi et al., 2020; Wang et al., 2022).

### 2.10 OM-LV20

OM-LV20 was identified from the skin secretion of odorous frog, *Odorrana margaretae* (Li et al., 2018). OM-LV20 accelerated the healing of in a full-thickness dermal wound model in mice. An *in vitro* experiment showed that OM-LV20 promoted wound healing by inducing keratinocytes (HaCaT) and HSF migration. Additionally, it induced the proliferation of HaCaT but not HSF cells. In addition, OM-LV20 showed no direct antimicrobial activity, hemolytic activity, or acute toxicity.

### 2.11 OA-GL21 and OA-GL17

OA-GL21 was isolated from the skin secretion of *Odorrana andersonii* (Bian et al., 2018). OA-GL21 significantly promoted wound healing of human keratinocytes (HaCaT) and human fibroblasts in a dose- and time-dependent manner. However, OA-GL21 had no significant effect on the proliferation of these two cell lines. OA-GL21 obviously promoted wound healing in the full-thickness skin wound model in mice in dose-dependent and scar-free manners. Further studies showed that OA-GL21 had no direct antibacterial, hemolytic, and acute toxic activity, and it had weak antioxidant activity but high stability. OA-GL17 was another wound-healing-promoting peptide isolated from *O. andersonii* (Zhang et al., 2022). It effectively enhanced the proliferation and migration of keratinocytes and accelerated the repair of skin full-thickness wounds and scald wounds in mice. Furthermore, it upregulated the expression of transforming growth factor-β1 and activated the TGF-β1/Smad signaling pathway by decreasing the level of miR-663a.

### 2.12 Brevinin-2Ta

Brevinin-2Ta (B-2Ta) was isolated from the skin secretion of the European frog, *Pelophylax* kl*. esculentus* with wound-healing-promoting activity (Liu et al., 2017). B-2Ta was also a member of AMPs. The antimicrobial and hemolytic assay revealed that B-2Ta showed broad antimicrobial activities against *S. aureus*, *E. coli*, and *Candida albicans* with low cytotoxicity to erythrocytes. B-2Ta effectively promoted wound closure and restrained the bacterial infection in *Klebsiella pneumoniae*-infected wounds in SD rats. The histological study showed that B-2Ta improved skin re-epithelization, granulation, and collagen deposition in the wound area and enhanced epithelial migration and angiogenesis. On the cellular level, reduction in IL-10 demonstrated that B-2Ta could relieve inflammation by inhibiting the bacterial infection. These results indicated that B-2Ta reduced the continuous phase of inflammation and accelerated wound healing in *K. pneumonia*-infected rats via modulating re-epithelialization.

### 2.13 Brevinin-2PN

Brevinin-2PN is an antimicrobial peptide identified from *Pelophylax nigromaculatus* and showed potent antibacterial activity by destroying the bacterial membrane and targeting bacterial genomic DNA (Fan et al., 2022). In addition, brevinin-2PN also showed wound-healing-promoting activity. It effectively promoted the healing of HSF cell scratches by inducing cell migration and regulating the expression of growth factors.

### 2.14 Pse-T2

Pse-T2 is a truncated analog of pseudin-2, which was primarily isolated from the skin of the frog *Pseudis paradoxa* (Kang et al., 2018). Pseudin-2 is a 24-amino acid AMP that exhibits potent antibacterial activity against Gram-negative bacteria, but its cytotoxicity limited the application in clinic. As mentioned previously, it is more likely to modify a known peptide to enhance activity and reduce toxicity. The authors next assayed the activities of 14 modified peptides based on pseudin-2 (Kang et al., 2018). The data indicated that pseudin-2-truncated analogs (particularly Pse-T2) were the most effective antimicrobial agents against pathogenic bacteria in the modified peptides and exhibited little hemolytic activity. The *in vivo* assay showed that Pse-T2 also enhanced *P. aeruginosa*-infected wound closure via its antibacterial and anti-biofilm activities.

### 2.15 Tylotoin

Tylotoin was identified from the skin of salamander *Tylototriton verrucosus* with potential wound-healing-promoting activity (Mu et al., 2014). Tylotoin was a member of the cathelicidin antimicrobial peptide family, but it had no antimicrobial activities against tested bacteria. Interestingly, it showed comparable wound-healing-promoting ability with EGF in a murine model of full-thickness dermal wounds. Further research revealed that tylotoin directly enhanced the motility and proliferation of keratinocytes, proliferation of vascular endothelial cells and their tube formation, and proliferation of fibroblasts. Tylotoin significantly accelerated re-epithelialization and granulation tissue formation in the wound site. Tylotoin also promoted the release of TGF-β1 and IL-6, which were essential for wound healing.

### 2.16 TK-CATH

TK-CATH was identified from the salamander skin *Tylototriton kweichowensis*, which belonged to the cathelicidin antimicrobial peptide family (Luo et al., 2021). Intriguingly, TK-CATH is an anionic cathelicidin antimicrobial peptide that lacks direct antimicrobial activity. However, it effectively accelerated the healing of full-thickness skin wound in mice. It was demonstrated that TK-CATH elicited the production of cytokines, chemokines, and growth factors in that promoted cell migration, cell proliferation, and wound healing.

### 2.17 Ot-WHP

In our previous work, we also isolated a wound-healing-promoting peptide, designated as Ot-WHP, from Chinese concave-eared frog *Odorrana tormota* (He et al., 2019). In our study, Ot-WHP efficiently promoted wound healing in a murine model of full-thickness wounds. It significantly increased the number of neutrophils and macrophages in wound sites. However, our results showed that Ot-WHP did not act as a chemoattractant for neutrophils and macrophages. Then, we found that Ot-WHP significantly induced chemokine, cytokine, and growth factor production in macrophages by activating mitogen-activated protein kinases (MAPKs) and nuclear factor-kB (NF-kB) signaling pathways. It suggested that the chemotactic activity of Ot-WHP depended on inducing chemoattractant production in macrophages. In addition, Ot-WHP directly promoted keratinocyte migration by enhancing integrin expression and cell adhesion. Ot-WHP promoted the proliferation of keratinocytes and fibroblasts, collagen deposition, and α-SMA expression in fibroblasts via activating macrophages, which suggested that Ot-WHP significantly enhanced the crosstalk between macrophages and keratinocytes/fibroblasts, which, in turn, efficiently promoted wound closure in mice. Furthermore, Ot-WHP modestly promoted neutrophil phagocytosis and phorbol myristate acetate (PMA)-induced neutrophil extracellular trap formation, which were extremely critical for clearing off cell debris and microbes in wounds.

### 2.18 Bv8-AJ

Bv8-AJ was isolated from the skin secretion of *Amolops jingdongensis* and was identified as a homologous of mammalian prokineticins (Chang et al., 2019). Bv8-AJ significantly accelerated wound healing of full-thickness wounds in mice models. The histopathological study indicated that Bv8-AJ accelerated the initiation and cessation of the inflammatory phase of wounds in mice models. Moreover, Bv8-AJ exerted a strong proliferative effect on fibroblasts and keratinocytes isolated from newborn mice by activating the interleukin-1 signaling pathway.

### 2.19 DMS-PS2

DMS-PS2 was identified from the skin of the waxy monkey tree frog, *Phyllomedusa sauvagei* (Song et al., 2020). It was demonstrated to be a typical cationic antimicrobial peptide belonging to the dermaseptin family and have potent antibacterial activity against Gram-positive bacteria, Gram-negative bacteria, and fungi. DMS-PS2 also showed an inhibitory effect against Gram-positive and Gram-negative bacterial biofilms. Remarkably, DMS-PS2 effectively promoted the healing of methicillin-resistant *Staphylococcus aureus* (MRSA)-infected wounds in murine skin.

### 2.20 RL-10 and RL-QN15

RL-10 was identified from the skin of *Rana limnocharis* (Wang et al., 2021a). It significantly enhanced the migration and proliferation of HaCaT cells *in vitro*. RL-10 also effectively promoted the healing of a full-thickness wound in mice. RL-QN15 was another wound-healing-promoted peptide identified from the skin of *Rana limnocharis* (Wang et al., 2021b) and provided protection against chronic wounds, skin fibrosis, and oral ulcers. The mechanistic study revealed that RL-QN15 obviously induced the activation of MAPK and Smad signaling and selectively regulated the production of cytokines in macrophages.

### 2.21 FW-1 and FW-2

FW-1 and FW-2 were isolated from the skin of *Hyla annectans*, which were cleaved from one protein precursor containing five copies of FW-1 and four copies of FW-2 (Liu et al., 2021). FW-1 and FW-2 provided protection against ultraviolet B (UVB)-induced skin inflammation in mice. *In vitro*, they significantly inhibited inflammatory responses in UVB-irradiated keratinocytes, modulated the MAPK and NF-κB signaling pathways activated by UVB, and reduced the production of reactive oxygen species (ROS) induced by UVB.

### 2.22 PM-7

PM-7 was a wound-healing-promoting peptide isolated from the skin of *Polypedates megacephalus* (Fu et al., 2022). It efficiently promoted the healing of full-thickness skin wounds in mice and enhanced the proliferation and migration of HSF and HUVECs. In addition, it could induce the activation of MAPK signaling such as ERK and p38 in HUVECs.

## 3 Discussion and conclusion

We herein summarized amphibian-derived wound-healing-promoting peptides, which were mainly identified from anuran (frogs and toads) amphibians (Table 1). We also compared the mechanisms of these peptides, such as effector cells and signaling pathways (Table 2). Among these peptides, most of them could induce cell proliferation, cell migration, and the production of growth factors related to wound healing. However, the research focus of each peptide was different, and not every amphibian-derived wound-healing-promoting peptide has exactly uncovered its mechanism of action. Taking MSI-1 for example, it improved wound closure in the *E. coli*-infected burn-wound model mainly by decreasing bacterial counts and relieving the systemic inflammatory response induced by local infection. In addition, the enhanced effects of Pse-T2 and brevinin-2Ta on bacteria-infected wound closure are attributed to their antibacterial activities, which are similar to the MSI-1 peptide. Although the antimicrobial activities of other peptides were not reflected in the process of wound healing, the antimicrobial properties of these peptides play important roles in the process of wound healing. Furthermore, microbial infection is usually associated with wound, and infection is a major factor that hinders wound healing (Demidova-Rice et al., 2012; Mangoni et al., 2016). Microbial infection is one of the most common factors that result in chronic wounds (Mustoe, 2004). Opportunistic pathogens, such as the Gram-negative bacteria *P. aeruginosa* or the Gram-positive bacteria *S. aureus*, are able to colonize skin wounds and form biofilms (Mancl et al., 2013; Vyas and Wong, 2016). It is difficult for the host to eradicate microbial infection when microbial cells aggregate in the wound and immobilize in an adhesive matrix of extracellular polymeric substances. The microbial biofilm usually results in the weak penetration of antibiotics or host clearance (Mustoe, 2004; Wolcott et al., 2010; Taylor et al., 2014). Unlike traditional antibiotics, AMPs can efficiently control microbial proliferation and modulate host’s immune response to a variety of biological or physical insults, and these properties make AMP good candidates for dealing with the challenge of infectious wounds. As mentioned previously, MSI-1, Pse-T2, and brevinin-2Ta promoted wound healing by inhibiting bacterial infection. Among the listed wound-healing-promoting peptides in Table 1, seven peptides belonged to AMPs or their derivatives. The dual antimicrobial and immunomodulatory activities of AMPs in infectious wound healing make them particularly attractive candidates, possibly superior to conventional antibiotics, for the local treatment of infected skin wounds (Do et al., 2014).

**TABLE 1:** Information of amphibian-derived wound-healing-promoting peptides.

Peptide	Amino acid sequence	Molecular weight (Da)	Modification	AMP	Source
AH90	ATAWDFGPHGLLPIRPIRIRPLCG	2657.2	**/**	Yes	*Odorrana grahami*
CW49	APFRMGICTTN	1210.43	**/**	No	*Odorrana grahami*
Temporins A and B	Ta: FLPLIGRVLSGIL-NH_2_	1397.77	N-terminal amidation	Yes	*Rana temporaria*
	Tb: LLPIVGNLLKSLL-NH_2_	1392.79			
Esculentin1a(1-21)NH_2_	GIFSKLAGKKIKNLLISGLKG-NH_2_	2184.72	One N-terminal amidation	Yes	*Rana esculenta*
Tiger17	WCKPKPKPRCH-NH_2_	1376.79	N-terminal amidation, one intramolecular disulfide bond	No	*Fejervarya cancrivora*
MSI-1	GIWKFLKKAKKFWK	1807.36	**/**	Yes	*Xenopus laevis*
Cathelicidin-OA1	IGRDPTWSHLAASCLKCIFDDLPKTHN	3038.5	**/**	Yes	*Odorrana andersonii*
Cathelicidin-NV	ARGKKECKDDRCRLLMKRGSFSYV	2845.9	One intramolecular disulfide bond	Yes	*Nanorana ventripunctata*
Antioxidin-NV	GWANTLKNVAGGLCKMTGAA	1963.30	/		*Nanorana ventripunctata*
Cathelicidin-DM	SSRRKPCKGWLCKLKLRGGYTLIGSATNLNRPTYVRA	4165.9	Intramolecular disulfide bond	Yes	*Duttaphrynus melanostictus*
OM-LV20	LVGKLLKGAVGDVCGLLPIC	1966.39	One intramolecular disulfide bond	No	*Odorrana margaretae*
OA-GL21	GLLSGHYGRVVSTQSGHYGRG	2188.0	/	No	*Odorrana andersonii*
OA-GL17	GLFKWHPRCGEEQSMWT	2092.38	/	No	*Odorrana andersonii*
Brevinin-2Ta	GILDTLKNLAKTAGKGILKSLVNTASCKLSGQC	3347.01	One intramolecular disulfide bond	Yes	*Pelophylax kl. esculentus*
Brevinin-2PN	GLMDSLKGLAATAGKTVLQGLLKTASCKLEKTC	3349.04	One intramolecular disulfide bond	Yes	*Pelophylax nigromaculatus*
Pse-T2	LNALKKVFQKIHEAIKLI-NH_2_	2106.6	N-terminal amidation	Yes	*Pseudis paradoxa*
Tylotoin	KCVRQNNKRVCK	1473.79	One intramolecular disulfide bond	No	*Tylototriton verrucosus*
TK-CATH	GGQDTGKEGETGKKKKSDNWFMNLLNKFLELIGLKEAGDDSEPFCFTCIFDMFSQ	6196.99	/	No	*Tylototriton kweichowensis*
Ot-WHP	ATAWDLGPHGIRPLRPIRIRPLCG	2666.12	**/**	No	*Odorrana tormota*
Bv8-AJ	AVITGACERDVQCGGGTCCAVSLWMQGLRICTPLGRQGENCHPASRKVPFAGLRLHNSCPCQSNLACKTLPNGKYQCMPS	8485.9	Five intramolecular disulfide bonds	No	*Amolops jingdongensis*
DMS-PS2	ALWKTLLKNVGKAAGKAVLNAVTDMVNQ-NH_2_	2953.52	N-terminal amidation	Yes	*Phyllomedusa sauvagei*
RL-10	RLFKCWKKDS	1310.58	/	No	*Rana limnocharis*
RL-QN15	QNSYADLWCQFHYMC	1906.71	One intramolecular disulfide bond	No	*Rana limnocharis*
FW-1	FWPLI-NH_2_	673.8	N-terminal amidation	No	*Hyla annectans*
FW-2	FWPMI-NH_2_	691.9	N-terminal amidation	No	*Hyla annectans*
PM-7	FLNWRRILFLKVVR	1860.32	/	No	*Polypedates megacephalus*

/, no modification.

**TABLE 2:** Mechanism of amphibian-derived wound-healing-promoting peptides.

Peptide	Effector cell type							Signaling pathway
	Immunocyte		Keratinocyte		Fibroblast			
	Recruitment	Cytokine	Proliferation	Migration	Proliferation	Migration	Differentiation	
AH90	**/**	**+**	**-**	**+**	**/**	**/**	**+**	MAPK, NF-κB, Smad
CW49	**+**	**+**	**/**	**/**	**/**	**/**	**/**	HIF-1, eNOS and iNOS
Temporins A and B	**+**	**/**	**+**	**+**	**/**	**/**	**/**	**/**
Esculentin1a(1-21)NH_2_	**/**	**/**	**-**	**+**	**/**	**/**	**/**	STAT3
Tiger17	**+**	**+**	**+**	**+**	**+**	**/**	**+**	MAPK, Smad
MSI-1	**/**	**+**	**/**	**+**	**/**	**/**	**/**	**/**
Cathelicidin-OA1	**+**	**+**	**+**	**-**	**-**	**+**	**/**	**/**
Cathelicidin-NV	**/**	**+**	**+**	**+**	**+**	**/**	**+**	MAPK
Antioxidin-NV	**/**	**+**	**/**	**/**	**/**	**/**	**+**	TLR4/p38/JNK/NF-κB, TGF-β1/Smad2
Cathelicidin-DM	+	/	+	+	+	+	+	MAPKs
OM-LV20	**/**	**/**	**+**	**+**	**-**	**+**	**/**	**/**
OA-GL21	**/**	**/**	**-**	**+**	**-**	**+**	**/**	**/**
OA-GL17	**/**	**/**	**+**	**+**	**/**	**/**	**/**	miR-663a/TGF-β1/Smad axis
Brevinin-2Ta	**/**	**+**	**/**	**/**	**/**	**/**	**/**	**/**
Brevinin-2PN	**/**	**/**	**/**	**/**	**/**	**+**	**/**	/
Pse-T2	**/**	**+**	**/**	**/**	**/**	**/**	**/**	**/**
Tylotoin	**+**	**+**	**+**	**+**	**+**	**/**	**+**	MAPK, Smad
TK-CATH	/	+	+	+	/	/	/	MAPK
Ot-WHP	**+**	**+**	**+**	**+**	**+**	**/**	**+**	MAPKs, NF-κB, Smad
Bv8-AJ	**/**	**+**	**+**	**/**	**+**	**/**	**/**	IL-1/MAPK
DMS-PS2	**/**	**/**	**/**	**/**	**/**	**/**	**/**	/
RL-10	**/**	**/**	**+**	**+**	**/**	**/**	**/**	/
RL-QN15	**/**	**+**	**+**	**+**	**/**	**/**	**/**	MAPK, Smad
FW-1/FW-2	**/**	**+**	**/**	**/**	**/**	**/**	**/**	MAPK, NF-κB
PM-7	**/**	**/**	**/**	**/**	**+**	**+**	**/**	MAPK

/, no mentioned; -, had no effect; +, had positive effect.

The molecular weights of all the amphibian-derived wound-healing-promoting peptides are less than 3,500 Da (Table 1). Small molecular weight corresponds to low production cost, which makes the peptides possible to be widely applied in clinic. Meanwhile, the small molecular weight of peptide is more suitable for the preparation of emulsion ointment for the local treatment of skin wounds. As a topical agent, high local concentrations in the superficial soft tissues could be achieved easily, surpassing the concentrations that could be reached systemically (Mangoni et al., 2016). Among these peptides, we noticed that many of them were not natural peptides, but were modified by amidating the C-terminus known peptides or truncated from known peptides (Table 1). As we know, if the synthetic peptide is the internal sequence of one protein, the charge of peptide can be removed by N-terminal acetylation or C-terminal amidation, which will make it easy to maintain the natural structure and enhance the resistance of peptide to external peptidase. In addition, disulfide bonds between two cysteine residues can make peptide cyclization, which is usually an important way to improve the stability of one peptide. In addition, MSI-1 and Pse-T2 were truncated peptides of pexiganan and pseudin-2, respectively, which exhibited better activities and less toxicity. These provide efficient strategies to improve the activity and reduce the toxicity of known wound-healing-promoting peptides.

A series of wound-healing-promoting peptides have been characterized from amphibians, but these peptides had different research foci. Overall, the effects of most amphibian-derived wound-healing-promoting peptides on epithelial cells and immune cells were studied. In addition, some of them were studied on one special focus, such as CW49. The investigation of CW49 mainly focused on its pro-angiogenesisis effect in the wound-healing process and the related signaling pathways. Although different peptides play different roles in wound healing, these amphibian-derived peptides, in general, exhibited high efficiency in promoting wound healing in animal models. Considering that frog and mammalian skin share the same evolutionary ancestry, basic architecture, and conserved mechanisms underlying wound healing (Yoshizato, 2007), it is interesting to find more wound-healing-promoting peptides from amphibians, and the amphibian-derived wound-healing-promoting peptides might also be capable of promoting human skin wound healing and being selected as excellent templates for developing novel wound-healing-promoting agents in future.
